# Research on the Infiltration Effect of Waterborne Polyurethane Cementitious Composite Slurry Penetration Grouting Under Vacuum Effect

**DOI:** 10.3390/polym17233205

**Published:** 2025-12-01

**Authors:** Chungang Zhang, Feng Huang, Yingguang Shi, Xiujun Sun, Guihe Wang

**Affiliations:** 1 School of Engineering and Technology, China University of Geosciences, Beijing 100083, China; zhangcg1985@126.com (C.Z.); 3002240017@email.cugb.edu.cn (Y.S.); sxj140921@163.com (X.S.);; 2 Zhongkan Metallurgical Investigation Design & Research Institute Co., Ltd., Baoding 071069, China

**Keywords:** waterborne polyurethane, penetration grouting, vacuum action, percolation effect

## Abstract

To address the issue of restricted grout diffusion caused by seepage effects during grouting in sandy soil layers, this study proposes an optimised grouting method for water-based polyurethane-cement composite grout (WPU-CS) under vacuum-pressure synergy. By establishing a porous medium flow model based on the mass conservation equation and linear filtration law, the influence mechanism of cement particle seepage effects was quantitatively characterised. An orthogonal test (L_9_(3^4^)) optimised the grout composition, determining the optimal parameter combination as the following: water-to-cement ratio 1.5:1, polyurethane-to-cement ratio 5~10%, magnesium aluminium silicate content 1%, and hydroxypropyl methylcellulose content 0.15%. Vacuum permeation grouting tests demonstrated that compared to pure cement slurry, WPU-CS reduced filter cake thickness by 80%, significantly suppressing the leaching effect (the volume fraction δ of cement particles exhibited exponential decay with increasing distance r from the grouting end, and the slurry front velocity gradually decreased). Concurrently, the porosity ϕ in the grouted zone showed a gradient distribution (with more pronounced porosity reduction near the grouting end). When vacuum pressure increased from −10 kPa to −30 kPa, slurry diffusion distance rose from 11 cm to 18 cm (63.6% increase). When grouting pressure increased from 20 kPa to 60 kPa, diffusion distance increased from 8 cm to 20 cm (150% increase). The study confirms that synergistic control using WPU-CS with moderate grouting pressure and high vacuum effectively balances seepage suppression and soil stability, providing an innovative solution for efficient sandy soil reinforcement.

## 1. Introduction

Underground engineering construction encounters many karst and adverse geological problems, especially when crossing the Quaternary sand layer; it is very easy to induce geological disasters such as pipe surge, sand flow, and landslide [1]. The sandy soil layer has the characteristics of poor self-stability, uneven permeability, and tortuous pore channels, so the reinforcement of the sandy soil layer has become a global technical problem in the field of engineering. The grouting method is widely used in the reinforcement of porous media by virtue of its advantages of low engineering cost, avoiding secondary disasters, blocking hydraulic channels, and fixed-domain reinforcement of sandy-soil media. Permeation grouting involves injecting grout under controlled pressure into porous media, displacing resident air and water. Subsequent grout solidification achieves ground reinforcement and hydraulic sealing. During grout permeation, filtration effects arise as skeletal particles retain suspended grout solids through non-uniform deposition. This process attenuates grout propagation distance and induces progressive weakening of consolidation efficacy along the flow path [2,3]. Filtration effects inherently occur during grout permeation through porous media. To mitigate their adverse impacts on grouting efficiency, there are escalating demands for enhanced grout material properties and technological advancements. Waterborne polyurethane cementitious composite slurry has been widely used in sand infiltration grouting projects due to its environmentally friendly, chemically resistant, and fast-curing characteristics.

In recent years, extensive theoretical and experimental research has been conducted on the migration-filtration mechanism of suspended particles within porous media, focusing on the permeation patterns of grout. Early studies concentrated on establishing theoretical models for grout diffusion and filtration. Feng [4] developed a grouting model applicable to gravel formations to elucidate the diffusion and solidification mechanisms of grout. Subsequently, F. Bouchelaghem et al. [5] achieved simulation of permeation grouting within deformable saturated porous media, incorporating both slurry migration and particle filtration processes. To quantitatively characterise this phenomenon, Bradford et al. [6] and Bedrikovetsky et al. [7], respectively, proposed models describing suspended particle adsorption and filtration, introducing irreversible first-order filtration coefficients. Through cement slurry grouting experiments, Saada et al. [8] developed a theoretical model for grouting in granular media incorporating seepage effects, comparing analytical and numerical solutions. Poruban et al. [9] further distinguished between the seepage behaviour of spherical and non-spherical particles, noting that the continuous accumulation of particles within pore spaces leads to a gradual attenuation of seepage rates. Experimentally, Wang et al. [10] developed a specialised grouting apparatus to systematically analyse the control exerted by grouting parameters and sandy soil properties over slurry diffusion behaviour, providing empirical support for the theoretical framework.

Substantial research efforts have also addressed vacuum-assisted permeation grouting. Scholars have advanced this domain primarily through experimental and theoretical frameworks. Experimentally, researchers including Jiang [11], Wang [12], Zhang [13], and Shen [14] conducted laboratory-scale physical modelling to validate vacuum grouting efficacy. Their results demonstrate that vacuum field application not only extends the grout propagation range but also enables directional transport manipulation. Further investigations by Li [15] into unsaturated cohesive soils, and by Lyu et al. [16] considering porous media flow effects, have elucidated the grout diffusion mechanisms and the factors influencing the mechanical properties of the solidified grout body. Theoretically, efforts have been focused on refining diffusion models. Based on the mechanisms of vacuum preloading and grouting theory, Huang [17] corrected the boundary conditions of the Maag spherical diffusion model, leading to a vacuum-modified model. Building on this, Gao [18] derived formulas describing the distribution of vacuum pressure and further revised the theoretical model for grout diffusion under vacuum negative pressure, incorporating principles of permeation grouting and a planar seepage flow model. A consensus emerging from these studies is that the vacuum negative pressure significantly increases the grout penetration radius. Furthermore, it has been established that this radius exhibits a positive correlation with both the permeability coefficient of the stratum and the magnitude of the applied vacuum.

Both domestic and international research have also explored the role of polymers in enhancing the properties of cementitious grouting materials. Regarding mechanical performance, polyacrylate emulsions [19] and self-synthesised anionic water-based polyurethanes [20] have been demonstrated to improve the mechanical behaviour of cementitious materials. However, Çolak A et al. [21] noted that combining VAE with high-efficiency water-reducing agents reduces the mechanical strength of mortar. Fan et al. [22] and Hisona et al. [23], respectively, developed polyurethane-cement composite grouting materials and systematically evaluated their comprehensive performance. Regarding durability and microstructural mechanisms, research generally agrees that polymers optimise pore structure and enhance durability. Ramli et al. [24] observed linear relationships between permeability and compressive strength of polymer-modified mortars and curing humidity. Barluenga G et al. [25] indicated that polymer emulsions reduce macropore content and enhance resistance to ion permeation; building upon this, Liu et al. [26] developed novel grouting materials combining high impermeability with strength. Microscopic observations further elucidated the mechanism as follows: Kong et al. [27] combined thermal analysis with in situ XRD, revealing that cement hydration is influenced by the surface charge of polymer particles, with anionic emulsions exhibiting more pronounced inhibitory effects. Acid leaching tests and SEM observations by Zhang et al. [28] corroborated the formation of polymer films within styrene-acrylic emulsion-modified mortar, consistent with the dual-network structure proposed by the Konietzko model [29]. Piapanou et al. [30] similarly confirmed via SEM that polymer films at interfaces effectively enhance mortar bonding properties.

Current research on infiltration grouting primarily focuses on atmospheric pressure conditions, while vacuum-assisted grouting studies have been predominantly concentrated on cementitious or chemical slurries. Notably, the infiltration behaviour of WPU-CS under vacuum remains unreported. This study systematically investigates the infiltration grouting performance of WPU-CS. Through a series of proportioning tests, we first determined the optimal mix ratio of the composite slurry. Subsequently, infiltration grouting tests under vacuum conditions were conducted to elucidate the influence of vacuum degree and grouting pressure on the grouting effect, including the infiltration effect, grouting volume, and slurry diffusion distance. The synergistic application of WPU-C composite grout with vacuum-assisted technology establishes an optimised methodology for weak formation permeation grouting. This approach effectively mitigates diffusion attenuation induced by filtration effects in conventional single-component grouts, providing a technical pathway for precision-controlled grouting processes.

## 2. Theoretical Model

**Figure 1:**
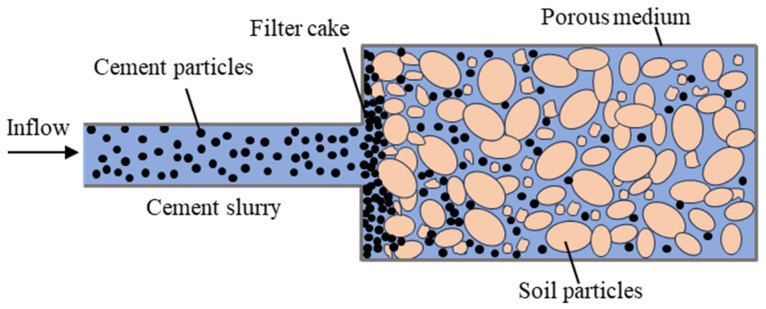
The filtration effect schematic diagram.

When the deposition of cement particles occurs, the closer the area is to the grouting end, the more significant the filtration effect is, and the more particles are deposited, while the farther the area is from the grouting end, the amount of cement particles deposited is reduced accordingly, as shown in Figure 1.

When the cement slurry penetrates the porous medium soil, the soil consists of soil particles, cement particles, and water. Let $\phi_{s}$, $\phi_{c}$, and $\phi_{w}$ be the volume fraction of soil particles, cement particles, and water in the soil, respectively. Therefore, the volume fraction of pore space, i.e., porosity $\phi =\phi_{w}+\phi_{c}$, is related to the volume fraction of soil particles in the skeleton, i.e., $\phi =1-\phi_{s}$. The hydromechanical dispersion and diffusion are not considered. This is consistent with a large number of experimental observations, which commonly attribute to the filtration phenomenon the dominating role during the injection process of grout injection in porous media [31]. Therefore, the macroscopic velocities ${\underline{v}}^{w}$ and ${\underline{v}}^{c}$ of water and cement particles are considered equal, i.e., ${\underline{v}}^{w}={\underline{v}}^{c}=\underline{v}$; in addition to this, it is assumed that the solid skeleton of the soil body is rigid, and it does not change in the process of grouting. For instance, Bouchelaghem et al. [32] obtained strains of the order of 10^−6^. At least in such a situation, the injection process does not induce significant irreversible rearrangement of the granular structure. In order to study the percolation effect of the slurry, this phenomenon is considered through the mass exchange between the cement particles and the skeleton particles. More precisely, −μ denotes the mass rate of cement filtered through the solid skeleton per unit volume. It can be seen from the analysis that −μ will increase with the volume fraction of cement, and −μ=0 when $\phi_{c}\to 0$. For a given ϕ, h(δ) it will decrease with the increase in soil porosity ϕ. Let δ be the volume fraction of cement particles during grouting, i.e., $\delta =\phi_{c}/\phi$, based on the law of linear filtration [8]:

(1) $$\frac{\mu }{\rho_{c}}=h(\delta )$$

When δ=0 and h(0)=0 Taylor series expansion of Equation (1) and simplification yield:

(2) $$\frac{\mu }{\rho_{c}}=\lambda \delta =\lambda \frac{\phi_{c}}{\phi }$$

In the equation, μ denotes the mass rate of cement filtered through the solid skeleton per unit volume, g·ml^−1^·s^−1^; $\rho_{c}$ denotes the density of cement particles in g·ml^−1^; λ represents the filtration coefficient, s^−1^. The filtration coefficient quantifies the extent to which slurry particles are filtered and deposited within the pores of porous media such as soil and rock. λ characterises the strength of the filtration effect exerted by the medium on the particle slurry. For instance, the greater the λ value, the stronger the adsorption and interception effect of the medium’s framework on suspended particles, resulting in an increased filtration yield of the suspended particles; ϕ indicates the porosity of the soil medium, which is a dimensionless quantity; δ signifies the cement particle content in the slurry within the pores, which is also a dimensionless quantity.

Cement particle retention deposition is responsible for the reduction in pore space in porous media. In particular, the volume fraction of pore space with porosity ϕ due to percolation is decreasing. In turn, the reduction in the pore space causes a decrease in the intrinsic permeability coefficient. $K^{\prime }$, within the framework of micro- and macro-methods, and theoretically, the exact derivation of the latter is a function of the morphology of the pore space [33]. As for the description of the mass exchange parameters, we consider that the intrinsic permeability is related to the reduction in the porosity. In this paper, we use the expression for the change in permeability coefficient caused by the filtration effect [8]:

(3) $$K^{\prime }=\frac{K_{0}^{\prime }}{1+b(\phi -\phi_{0})}$$

In Equation (3), ϕ and $\phi_{0}$ represent the soil porosity and initial porosity, respectively. b is a negative scalar, indicating that the permeability coefficient is positively correlated with porosity. It reflects the sensitivity of the permeability coefficient to changes in porosity, typically ranging from −0.1 to −0.5 for sandy soils. $K^{\prime }$ and $K_{0}^{\prime }$ denote the soil permeability coefficient and the initial permeability coefficient, respectively, in cm/s.

**Figure 2:**
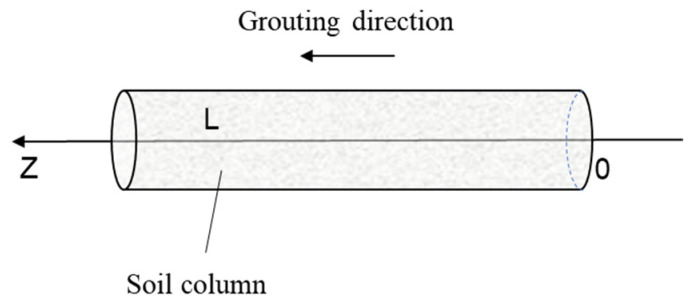
One-dimensional diffusion schematic diagram.

In this paper, a one-dimensional soil column is used for the study, where the cement slurry is injected into a vertical column of sandy soil. L denotes the height of the soil column, as shown schematically in Figure 2.

Seepage continuity equation for water based on engineering fluid dynamics:

(4) $$\frac{\partial (\rho_{w}\phi_{w})}{\partial t}+div(\rho_{w}\phi_{w}v)=0$$

Equation (5) is the mass balance equation for cement particles, which contains a source term that accounts for the mass exchange between cement particles and solid particles.

(5) $$\frac{\partial (\rho_{c}\phi_{c})}{\partial t}+div(\rho_{c}\phi_{c}\underline{v})=\mu$$

Since the solid soil skeleton is assumed to be rigid, the same source terms (with opposite signs) are in the mass balance equation for solid particles as follows:

(6) $$\frac{\partial (\rho_{s}\phi_{s})}{\partial t}=-\mu$$

In Equation (6), $\rho_{s}$ denotes the density of solid particles. Given the comparable densities of cement particles ($\rho_{c}$) and soil particles ($\rho_{s}$), the variation in $\rho_{s}$ is considered negligible. Thus, Equation (6) simplifies to:

(7) $$\frac{\partial (\phi_{s})}{\partial t}\approx -\frac{\mu }{\rho_{s}}\approx -\frac{\mu }{\rho_{c}}$$

Equation (7) expresses the relationship between the change in porosity and the mass exchange coefficient μ in the soil. Coupling Equations (2) and (7) gives the relationship between the change in porosity and the volume fraction of cement particles in the pores:

(8) $$\frac{\partial (\phi )}{\partial t}=\frac{\mu }{\rho_{c}}=\lambda \delta$$

Mass balance equations for cement and water at the bottom (grouted end) of a sandy soil column:

(9) $$\left\{\begin{array}{c} fv^{-}=\phi_{c}(0,t)v^{+}(t)\\ \left(1-f)v^{-}=\phi_{w}(0,t)v^{+}(t\right) \end{array}\right.$$

Equation (9), where f denotes the cement volume fraction in the grout before injection, $\nu^{-}$ represents the velocity of water and cement particles before entering the sandy soil (independent of time), and $\nu^{+}(t)$ denotes the velocity of water and cement particles upon entering the porous medium. Adding the two expressions in Equation (9) yields:

(10) $$\phi (0,t)\nu^{+}(t)=\nu^{-}$$

In turn, Equations (9) and (10) provide boundary conditions for the cement particle content δ of the slurry in the pores, at the grouting end:

(11) $$\delta (0,t)=\frac{\phi_{c}(0,t)}{\phi (0,t)}=f\;\;\forall t\geq 0$$

The porosity of a porous medium filled with water is homogeneous, i.e., the initial conditions:

(12) $$\left\{\begin{array}{l} \phi (0\ll z\ll l,t=0)=\phi_{0}\\ \delta (0\ll z\ll l,t=0)=0 \end{array}\right.$$

The intrinsic densities of cement particles and water are constant, so the mass Equations (4) and (5) can be expressed as:

(13) $$\frac{\partial (\phi_{w})}{\partial t}+div(\phi_{w}v)=0$$

(14) $$\frac{\partial (\phi_{c})}{\partial t}+div(\phi_{c}\underline{v})=\frac{\mu }{\rho_{c}}$$

This can be deduced by adding Equations (13) and (14) and relying on Equation (8):

(15) $$div(\phi \underline{v})=0$$

Combining Equations (10) and (15), it can be deduced that the

(16) $$\phi (z,t)v^{+}(z,t)=\phi (0,t)v^{+}(t)=v^{-}\;\forall t\geq 0,\forall z\in [0,L]$$

Associations (8), (14), and (16) are obtained:

(17) $$\phi \frac{\partial (\delta )}{\partial t}+\nu^{-}\frac{\partial (\delta )}{\partial z}=-\lambda \delta (1-\delta )$$

Equation (17) is the basic equation for the transport of cement particles when percolation effects are considered, with initial and boundary conditions of Equations (11) and (12) and unknown variables of ϕ and δ.

To find the analytical solution of Equation (17), it is first assumed that the variation in the original porosity in the soil with time and space can be neglected in Equation (17). Considering the boundary conditions and initial conditions, Equations (11) and (12), the solution of the differential Equation (17) is:

(18) $$\delta (z,t)=\left\{\begin{array}{cc} \frac{1}{1+\left(\frac{1}{f}-1\right)e^{\frac{\lambda z}{\phi_{0}v_{0}^{+}}}} & Z\leq v_{0}^{+}t\\ 0 & Z>v_{0}^{+}t \end{array}\right.$$

Derived by substituting Equation (18) into Equation (7) and considering the initial condition Equation (12):

(19) $$\phi (z,t)=\left\{\begin{array}{cc} \phi_{0}-\frac{\lambda \left(t-\frac{z}{v_{0}^{+}}\right)}{1+\left(\frac{1}{f}-1\right)e^{\phi_{0}v_{0}^{+}\lambda z}} & Z\leq v_{0}^{+}t\\ \phi_{0} & Z>v_{0}^{+}t \end{array}\right.$$

By bringing Equation (19) into Equation (3), the change rule of the permeability coefficient of the soil body in the range of slurry permeability can be obtained, and the reason for the decrease in the slurry permeability rate can also be explained by the change in the permeability coefficient. Given the close relationship between porosity and this model, an analysis of the impact on porosity via the vacuum-pressure coupling mechanism is conducted, thereby investigating its influence on infiltration effects.

## 3. Materials and Methods

### 3.1. Materials

#### 3.1.1. Cement

**Figure 3:**
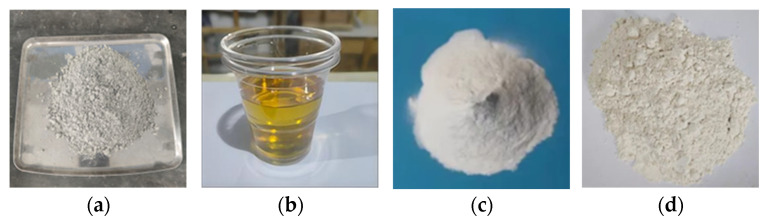
Test material. (**a**) Clinker; (**b**) Waterborne polyurethane; (**c**) Hydroxypropyl methyl cellulose; (**d**) Magnesium aluminium silicate.

**Figure 4:**
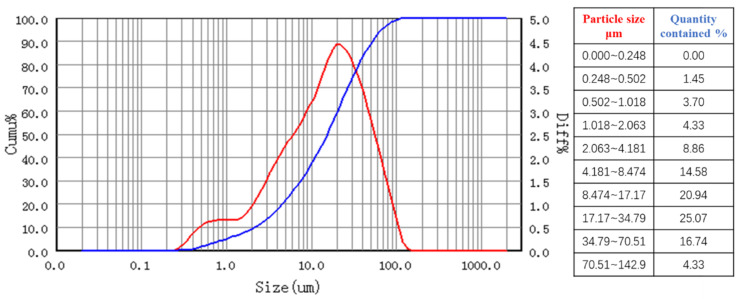
Grain size distribution curve.

Ultrafine silicate cement (Grade 42.5 R, Zhongde Xinya Co., Ltd., Zhengzhou, China) was employed in this study. The material morphology is characterised in Figure 3a, with key performance parameters quantified in Table 1. The cement was analysed by particle size analysis using the Bettersize2000 Laser Particle Size Distribution Meter (Bettersize Instruments Ltd., Dandong, China), and the particle size distribution curve is shown in Figure 4.

#### 3.1.2. Polyurethane

Waterborne polyurethanes are categorised into one-component and two-component systems. This study utilised a one-component aqueous polyurethane (commercial product code: YH-201), which was provided by Beijing Oriental Rainbow Waterproof Technology Co., Ltd., Beijing, China. As illustrated in Figure 3b, the material presents as a translucent yellowish liquid with a density of 1.1 g/mL. The pH ranges from 6.0 to 9.0, with a non-volatile content between 49% and 50%. Rheological characterisation indicates a viscosity range of 40–600 mPa·s, while thermal analysis reveals a minimum film-forming temperature of approximately 5 °C.

#### 3.1.3. Hydroxypropyl Methyl Cellulose

The hydroxypropyl methyl cellulose (HPMC) employed in this study was a 20 W viscosity grade supplied by Jinzhou Shunyue Building Material Science and Technology Co., Ltd., Shijiazhuang, China. As depicted in Figure 3c, the material presents as a white, non-toxic powder that readily dissolves in cold water to form a transparent, viscous solution. Detailed technical parameters are provided in Table 2. HPMC initially promotes early-stage hydration reactions; however, its elevated viscosity progressively impedes water transport to unreacted cement particles. This suppression of moisture diffusion attenuates late-stage hydration kinetics, resulting in prolonged final setting duration.

#### 3.1.4. Magnesium Aluminium Silicate

The aluminium magnesium silicate (designated as HD-201) utilised in this study is presented in Figure 3d. This material is synthesised from various natural nano-silicate minerals through purification and refinement processes that preserve the original crystalline structures, including flake and rod morphologies. Owing to its layered mineral configuration, HD-201 exhibits high hydrophilic characteristics, readily absorbing water to form macromolecular ionic hydrates between interlayer spaces. This unique structure endows the material with exceptional water retention and transport capacity. Based on the above functional properties, it can play an excellent water retention, thixotropic, anti-sinking, and emulsification performance in extremely harsh environments.

### 3.2. Method

#### 3.2.1. Grout Formulation Design and Test Specimen Fabrication

**Figure 5:**
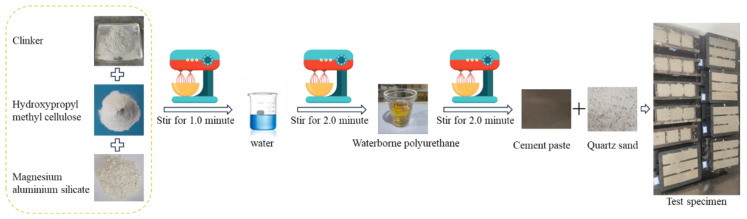
Slurry and test block making process.

**Figure 6:**
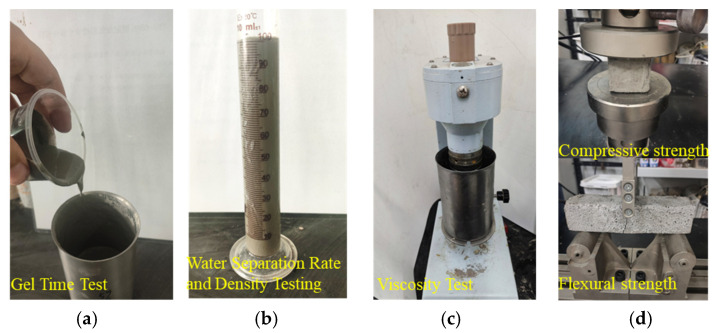
Slurry working performance and grouting, plus solid mechanical property test. (**a**) Gel Time Test; (**b**) Water Separation Rate and Density Testing; (**c**) Viscosity Test; (**d**) Compressive strength and Flexural strength.

To analyse the effects of water–cement ratio, poly–cement ratio, aluminium magnesium silicate (hereinafter referred to as suspending agent), and hydroxypropylmethylcellulose (hereinafter referred to as water-retaining agent) content on the working performance of waterborne polyurethane cement-based composite slurry, a four-factor, three-level orthogonal test L_9_ (3^4^) was used, as shown in Table 3. Table 3 and Table 4 shows the water–cement ratio as the weight ratio of water to cement, while the polymer-to-cement ratio, suspending agent, and water-retention agent are all expressed as percentages of cement mass, designated, respectively, as factors A, B, C, and D. This study investigates water-based polyurethane cementitious composite slurries, wherein cement remains the primary constituent. The water-based polyurethane serves solely to modify the cementitious slurry, necessitating its addition only in specific proportions—typically ranging from 5% to 15% [34]. Similarly, water-retaining agents and suspending agents require incorporation at corresponding ratios. The slurry was prepared by drying and stirring the cement, water-retaining agent, with the suspending agent for 1.0 min, and then slowly adding water and water-based polyurethane solution. The preparation process is shown in Figure 5, a total of 9 groups of slurry preparation, and the ingredients ratio is detailed in Table 4. This test was carried out using the inverted cup method for the gel time test, a six-speed rotational viscometer for the viscosity test, and a measuring cylinder for the precipitation rate and density test, as shown in Figure 6a–c.

The orthogonal test L_9_ (3^4^) was also used to prepare 9 sets of consolidated grout specimens. The volume ratio of slurry to quartz sand was 1:3, stirred well and put into moulds, the fabrication process was as shown in Figure 5, and finally, standard maintenance was carried out. The sizes of the specimens were 5 cm × 5 cm × 5 cm and 4 cm × 4 cm × 26 cm, respectively. Additionally, 14 d compressive strength and flexural strength tests were conducted on the specimens, as shown in Figure 6d.

#### 3.2.2. Infiltration Grouting Tests

1. Test equipment

**Figure 7:**
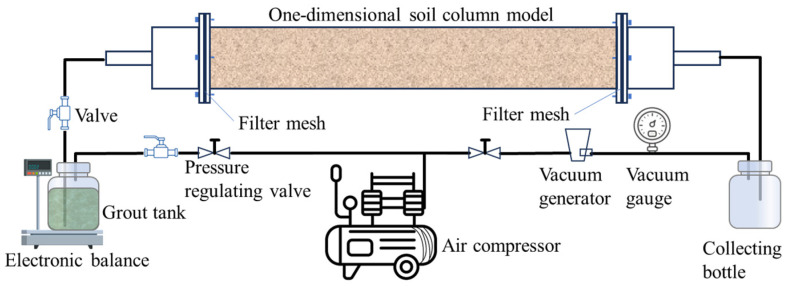
Schematic diagram of the test setup.

**Figure 8:**
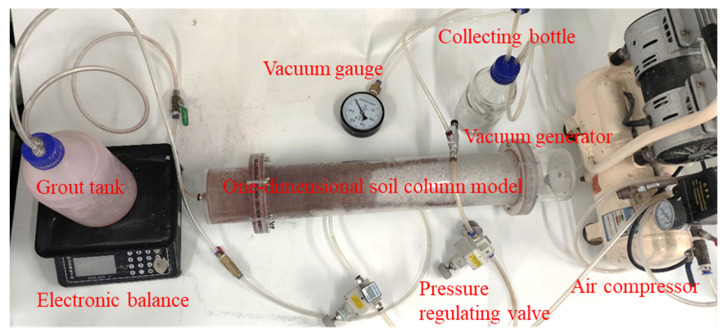
Infiltration grouting test.

The infiltration and diffusion behaviour of WPU-CS in sandy soil layers under vacuum conditions was investigated using a self-developed visual grouting simulation apparatus. As illustrated in Figure 7 and Figure 8, the experimental setup comprises three primary subsystems: a grouting system for precise slurry delivery, a transparent soil column model for observation of the infiltration process, and a vacuum generation system to simulate in situ negative pressure environments. The soil column model is made of acrylic with a length of 50 cm and a diameter of 10 cm, and the grouting system consists of an air compressor, a grouting pipe, a grouting bucket, a precision regulator, and an adapter. The vacuum generation system comprises an air compressor, vacuum generator, pressure regulator, and auxiliary components.

2. Pilot programme

Firstly, pure cement slurry was used for the penetration grouting test as a control group. Secondly, different vacuum (−10 kPa, −20 kPa, −30 kPa) and grouting pressure (20 kPa, 40 kPa, 60 kPa) conditions were selected for the composite slurry infiltration grouting test, and all of them used Group ④ composite slurry, and the grouting time was 100 s. The test programme is shown in Table 5. In this test, sand samples of 0.5 mm~1 mm size with porosity of 0.45 were used to record the grouting volume and measure the spreading distance of the slurry by observing the size of the filter cake thickness at the grouting end.

3. Test procedure

After connecting the test device, the experimental sand was deposited in five layers, each weighing 1.4 kg, and compacted sequentially. Following compaction, the sand sample heights were 10 cm, 20 cm, 30 cm, 40 cm, and 50 cm, respectively. This ensured consistent density across each layer, thereby guaranteeing repeatability and minimising experimental error. After assembly, carry out a vacuum test, and check the status of each instrument to ensure that the test device has good sealing. After the preparation of the slurry, add the red colouring agent, then pour it into the grouting bucket, weigh and record the total weight of the grouting bucket. A comparative test was conducted with and without the addition of red dye. The viscosity difference was less than 0.6%, and the density difference was merely 0.15%. Therefore, the impact of the red dye on the slurry’s rheology or density may be disregarded. Turn on the air compressor, adjust the regulator to change the vacuum degree of the vacuum generator, so that the vacuum gauge at the grouting position is stabilised at the predetermined vacuum degree (−10 kPa, −20 kPa, −30 kPa). Adjust the other regulator to stabilise the grouting pressure at a fixed value, open the valve so that the slurry is injected into the sandy soil, and record the grouting time and the weight shown on the electronic scale. Control the grouting time for 100 s, record the total weight of the grouting bucket after the end of grouting to obtain the amount of grout injected, and at the same time, measure the distance of grout penetration and diffusion. After 12 h, remove the sandy soil from the model, and carry out the next group of tests.

## 4. Results

### 4.1. Slurry Working Properties

**Figure 9:**
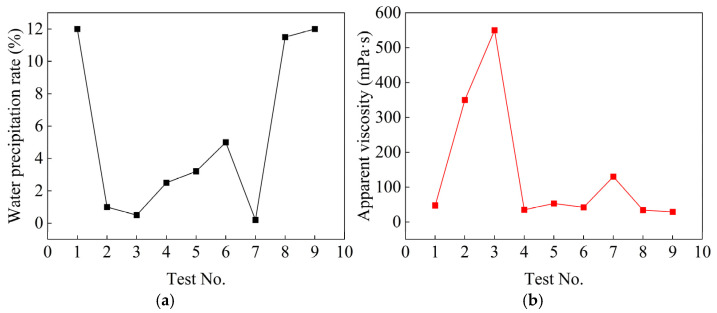

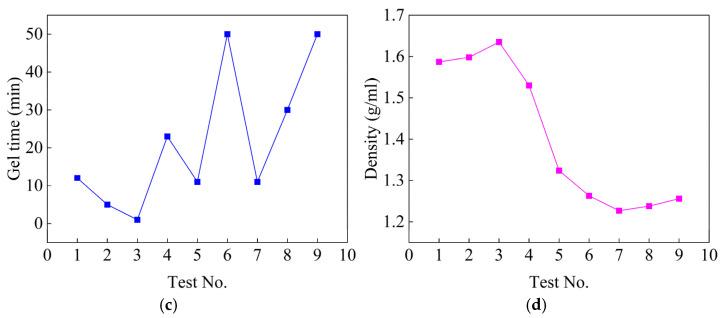
Composite slurry working performance test results. (**a**) Water precipitation rate; (**b**) Apparent viscosity; (**c**) Gel time; (**d**) Density.

Figure 9 shows the working performance test results of waterborne polyurethane cement-based composite slurry. From Figure 9, the water precipitation rate of 0.2~12%, a wide range of changes, and when the penetration grouting is performed, the water precipitation rate should be less than 5.0% [35]. Cement-based grout may be injected into soil layers with particle sizes ranging from 0.5 mm to 1 mm, with an appropriate viscosity range of 40 mPa·s to 47 mPa·s [36]. The apparent viscosity is 29 mPa·s minimum and 550 mPa·s maximum; again, smaller viscosity indicates the better fluidity of the slurry and the easier it is to inject into the soil body. The range of gel time is 1 min to 50 min; gel time is also critical for penetration grouting. According to the field construction experience, gel time should be controlled within 25 min. Figure 9 demonstrates an inverse correlation between slurry density and water–cement ratio. Increased water proportion reduces density, with mean values measuring 1.607 g·mL^−1^ (1:1), 1.372 g·mL^−1^ (1.5:1), and 1.240 g·mL^−1^ (2:1).

### 4.2. Mechanical Properties of Consolidated Grout Specimen

**Figure 10:**
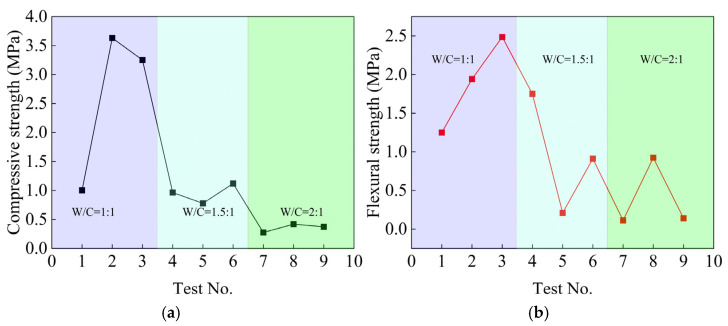
Compressive strength and flexural strength test results. (**a**) Compressive strength; (**b**) Flexural strength.

Figure 10 shows the test results of the 14-day compressive strength and flexural strength of the consolidated grout specimen. From Figure 10, the smaller the water–cement ratio is, the greater the compressive and flexural strength of the consolidated grout specimens are. The compressive and flexural strengths of consolidated grout specimens vary considerably with the slurry ratio, reaching maxima of 3.631 MPa and 2.486 MPa. In comparison, the minimum values are 0.274 MPa and 0.113 MPa.

### 4.3. Infiltration Test Result

#### 4.3.1. Effect of Vacuum Level on Diffusion Distance

**Figure 11:**
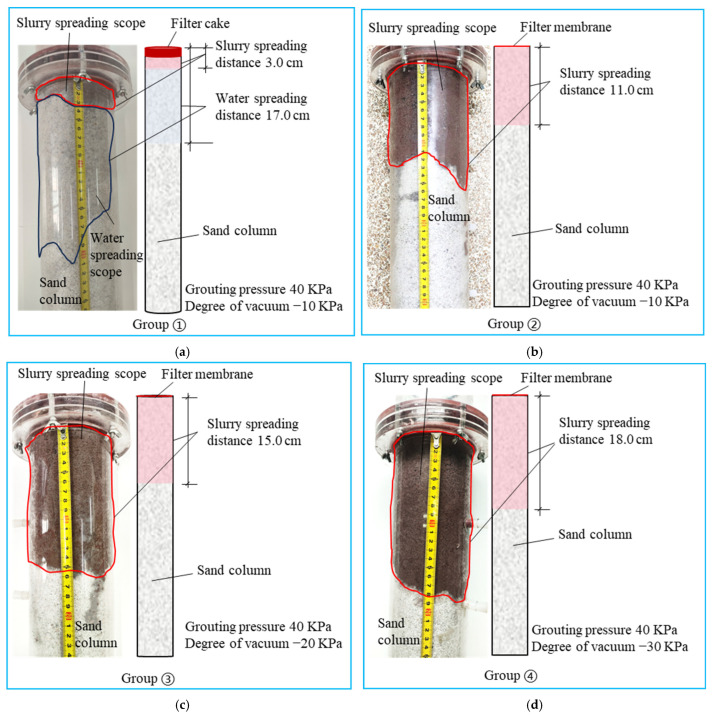
①~④ group infiltration grouting test results. (**a**) ① Group test results; (**b**) ② Group test results; (**c**) ③ Group test results; (**d**) ④ Group test results.

**Figure 12:**
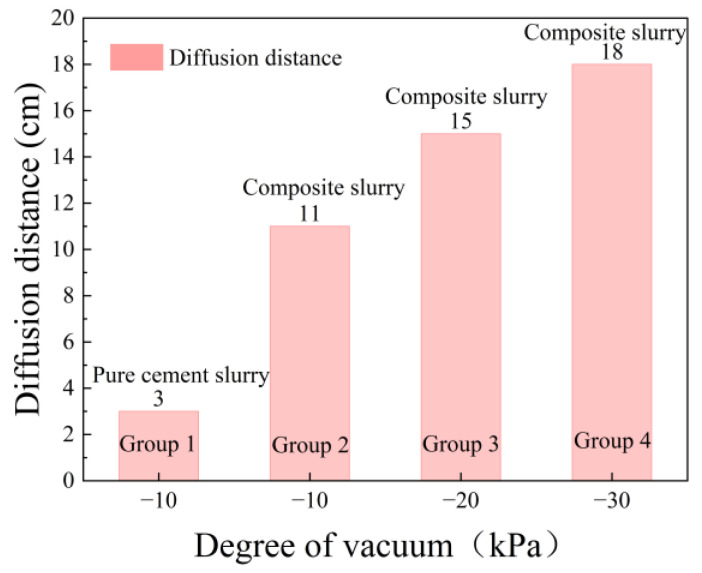
Slurry diffusion distance at different vacuum levels.

**Figure 13:**
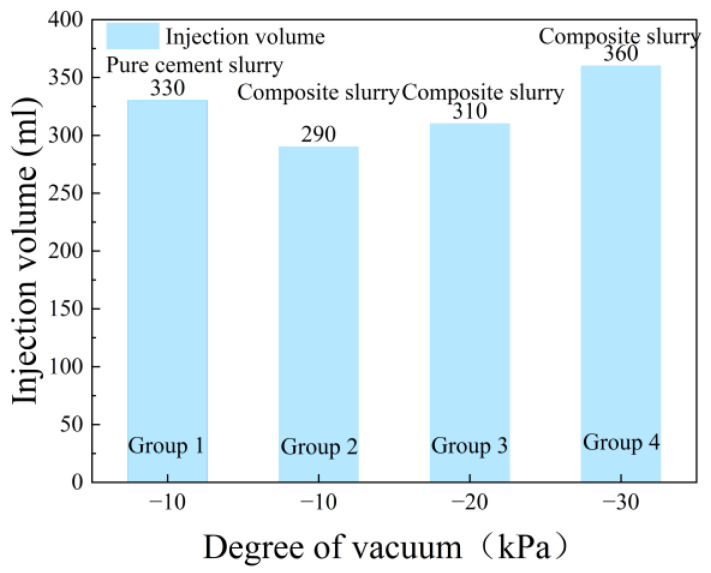
Injection volume at different vacuum levels.

**Figure 14:**
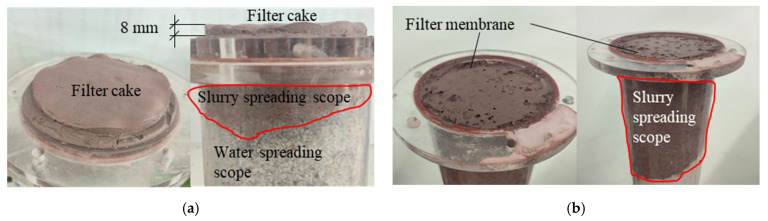
Filter cake at grouting end. (**a**) Pure cement slurry penetration grouting; (**b**) Composite slurry penetration grouting.

Figure 11 shows the results of the infiltration grouting test of group ①~④. Consistent with Figure 11a, neat cement grout injection (330 mL) yielded a restricted propagation distance of 3.0 cm, with rapid particle deposition forming a 0.8 cm filter cake. This pore clogging then induced a notable dehydration phenomenon over 17 cm. From Figure 11b–d, it can be seen that the dehydration phenomenon disappeared when the waterborne polyurethane cement-based slurry infiltrated the grouting. Figure 12 presents the grout propagation distances from permeation grouting tests (Groups ① to ④). The control group (①, pure cement grout) exhibited a limited penetration of 3.0 cm. In contrast, the composite grout used in Group ② achieved a significantly extended propagation distance of 11.0 cm, marking a 266.7% increase. When the vacuum pressure was elevated from −10 kPa (Group ②) to −30 kPa (Group ④), the propagation distance increased progressively from 11 cm to 18 cm, corresponding to a 63.6% enhancement. This trend clearly demonstrates that the grout propagation distance correlates positively with the applied vacuum intensity. The corresponding grout intake volumes under different vacuum levels are shown in Figure 13. The pure cement grout (Group ①) required an intake of 330 mL. Conversely, the composite grout in Group ②, despite its greater penetration, showed a 12.1% reduction in intake (290 mL). This suggests a more efficient filling mechanism or reduced filtrate loss for the composite grout. Furthermore, as the vacuum intensity increased from −10 kPa to −30 kPa across Groups ② to ④, the grout intake increased from 290 mL to 360 mL, a 24.1% rise. This confirms that the grout intake also exhibits a positive correlation with the vacuum pressure. Figure 14 shows the thickness of the filter cake at the grouting end. As can be seen from Figure 14, during grouting with pure cement slurry, the filter cake thickness at the injection end was 8 mm. In contrast, during permeation grouting with composite slurry, only a filter membrane approximately 1 mm thick formed at the injection end.

#### 4.3.2. Effect of Grouting Pressure on Spreading Distance

**Figure 15:**
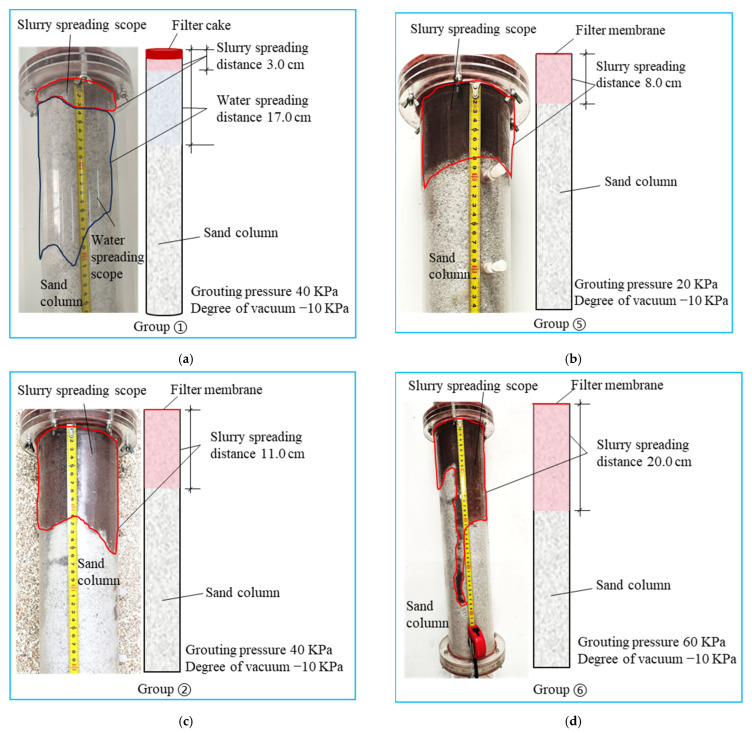
①, ②, ⑤, ⑥ group infiltration grouting test results. (**a**) ① Group test results; (**b**) ⑤ Group test results; (**c**) ② Group test results; (**d**) ⑥ Group test results.

**Figure 16:**
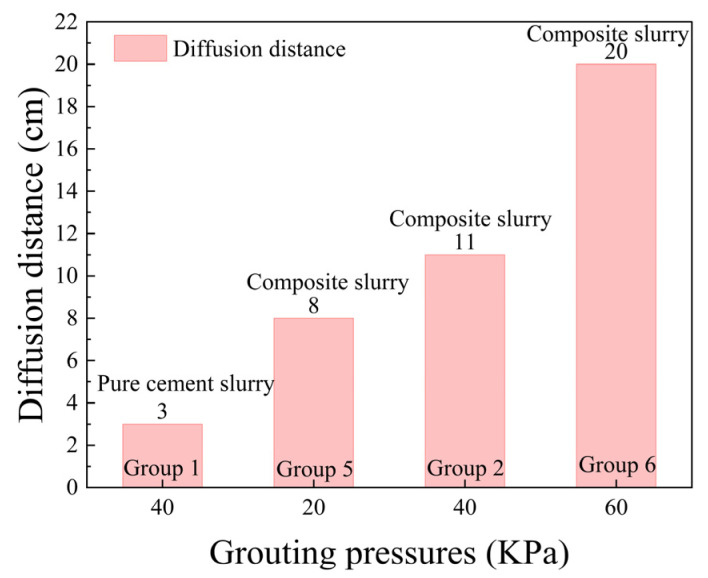
Slurry spreading distance at different grouting pressures.

Figure 15 shows the results of infiltration grouting tests for groups ①, ②, ⑤, and ⑥. Grouting pressure exhibits a positive correlation with infiltration distance in Figure 15. The measured distances progressively increase from 8 cm (20 kPa) to 11 cm (40 kPa) and 20 cm (60 kPa), representing a 150% total increase across the pressure range. The incremental increases in spreading distance reached 37.5% and 81.8% with rising pressure levels. Furthermore, Figure 15d reveals that under 60 kPa grouting pressure, localised slurry propagation attained 38 cm. This substantial extension suggests the development of enlarged pore channels within the sandy soil matrix under vacuum conditioning, thereby facilitating extensive slurry migration along these preferentially formed pathways under elevated grouting pressures. As shown in Figure 16, with the increase in grouting pressure, the slurry infiltration diffusion distance increases more rapidly.

## 5. Discussion

### 5.1. Analysis of Slurry Performance

**Figure 17:**
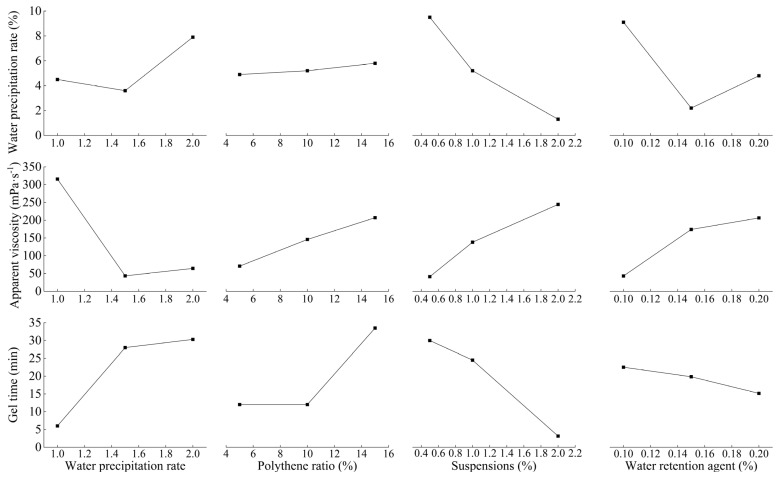
Plot of slurry performance indicators as a function of factors and levels.

Table 6 shows the extreme difference analysis table of the slurry working performance test results. From Table 6, the optimal solutions for water precipitation rate, apparent viscosity, and gel time are A_2_B_1_C_3_D_2_, A_2_B_1_C_1_D_1_, and A_1_B_1_C_3_D_3_, which are not the same as shown in Table 6. The variation in each index with the factor level was represented graphically as shown in Figure 17. The water–ash ratio is the most influential factor for apparent viscosity and gel time, and the best water–ash ratio is 1.5; for water precipitation rate, the best water–ash ratio is also 1.5. Poly–ash ratio for the three indicators is not the most influential factor, but all take 5% as the best. For the water precipitation rate and gel time, the suspending agent content of 2% viscosity is too large, and the overall consideration of the suspending agent content is 1%. Water-retention agent for precipitation rate is a minor factor; water-retention agent to take 0.15% is the best. For the apparent viscosity, the water-retention agent content of 0.1% is the best, followed by 0.15% is the next best; for the gel time, the water-retention agent content of 0.2% is the best, followed by 0.15%; for the three indicators of the comprehensive consideration, the water-retention agent content of 0.15% is the best. Based on the comprehensive balance method in orthogonal test, the optimal test programme A_2_B_1_C_2_D_2_ was obtained.

### 5.2. Analysis of Mechanical Properties of Grouting Plus Solids

**Figure 18:**
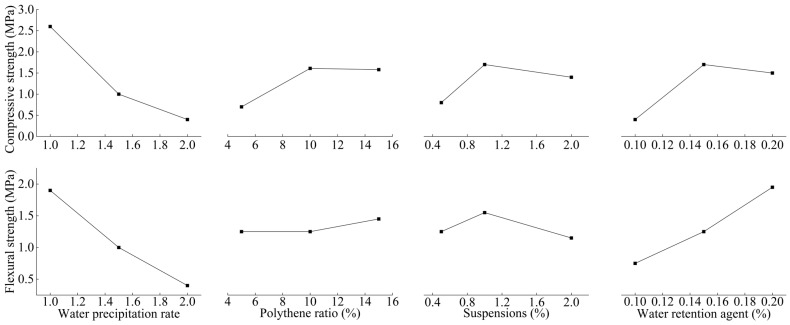
Plot of changes in injection stone body indicators as a function of factors and levels of change.

Table 7 shows the extreme difference analysis table for the test results of compressive strength and flexural strength. From Table 7, the optimal solutions are A_1_B_2_C_2_D_2_ and A_1_B_3_C_2_D_3_, respectively, as shown in Table 7. Plot the variation of each indicator with the factor level as shown in Figure 18. When Table 7 and Figure 18 are combined and analysed, the water–cement ratio is the most influential factor and it is best to take 1:1. Water–cement ratio of 1:1, compressive strength, and flexural strength are the largest, but the viscosity of the slurry and gel time is short, not suitable for infiltration grouting; after a comprehensive analysis, the water–cement ratio of 1.5:1. Water-retention agent is a less important factor; the overall consideration of the content of the water-retention agent to take the best of the 0.15 per cent. Poly–ash ratio is the least influential factor; taking 10% is best. Suspension agent content for compressive strength and flexural strength of the same law; take 1% as the best. Through the comprehensive analysis, the optimal scheme is A_2_B_2_C_2_D_2_. Finally, combined with Section 4.1, the optimal ratio of slurry is 1.5:1 water–cement ratio, 1% suspending agent, 0.15% water-retention agent, and 5%~10% poly–ash ratio.

### 5.3. Assessment of Filtration Effects

**Figure 19:**
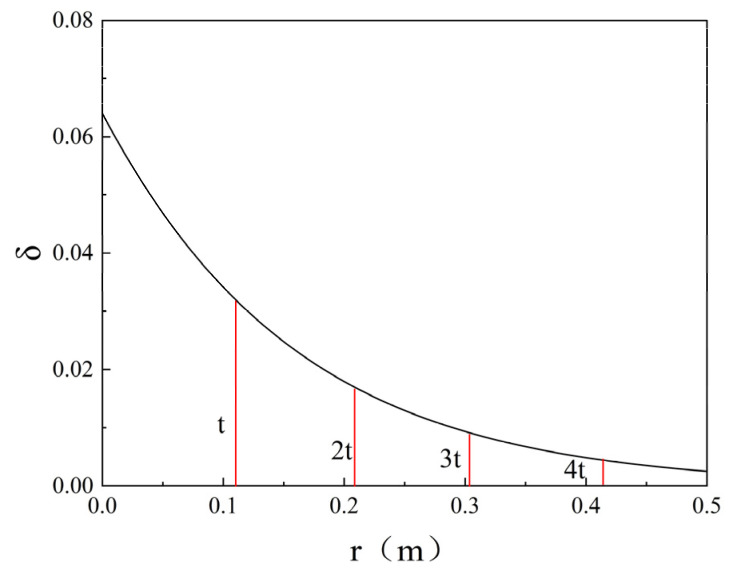
Profiles of the cement concentration at different times.

**Figure 20:**
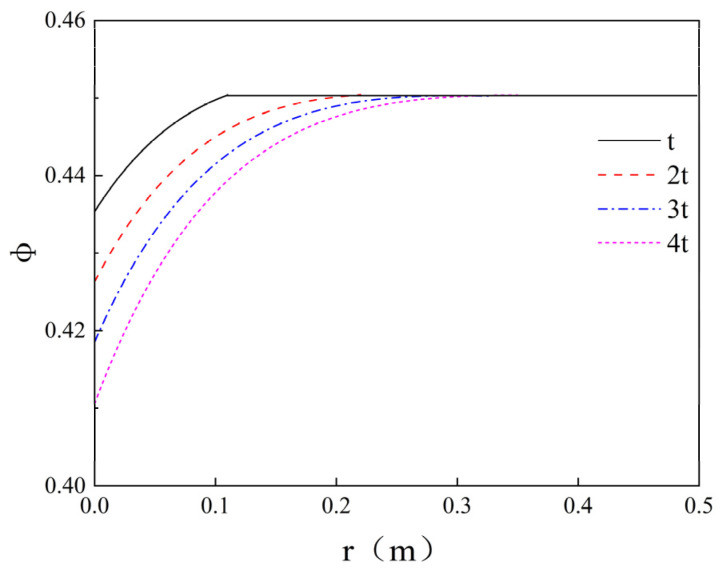
Profiles of porosity at different times.

To elucidate the governing mechanisms of filtration effects on grout permeation behaviour, according to Equations (18) and (19), the distribution of cement volume fraction δ and porosity ϕ at different times of infiltration grouting is plotted as shown in Figure 19 and Figure 20. The values of parameters are taken as f = 0.064, $\phi_{0}$ = 0.45, λ = 1.1 × 10^−3^ s^−1^, and $v_{0}^{+}$ = 0.369 × 10^−3^ m/s; λ values are referenced from existing infiltration coefficients tables [37]. Figure 19 illuminates the following two distinct behavioural regimes: (1) The volumetric particle concentration (δ) exhibits an inverse relationship with radial distance (r) from the injection point, attributable to progressive cement particle filtration. (2) The slurry front velocity demonstrates radial deceleration, as the unit volume of injected grout within the porous medium (2πrdz) increases proportionally with r during time increment dt. This velocity attenuation pattern aligns with the fundamental transport principles established in the theoretical framework of Maghous et al. [2]. The volume fraction of cement grout in porous media governs porosity variations. From Figure 20, it can be seen that the porosity decreases in the range of permeation grouting and decreases with increasing distance from the end of grouting, indicating the value of porosity reduction. Permeation grouting range gradually increases, the porosity within the grouting range gradually decreases, indicating that the cement particles in the cement slurry are retained in the pore space of the porous medium, resulting in a decrease in porosity. Equation (3) defines the soil permeability coefficient as an explicit function of porosity. Consequently, variations in porosity directly govern the magnitude of the permeability coefficient, which in turn dictates the efficacy of grout permeation during the injection process.

### 5.4. Analysis of the Effect of the Vacuum

**Figure 21:**
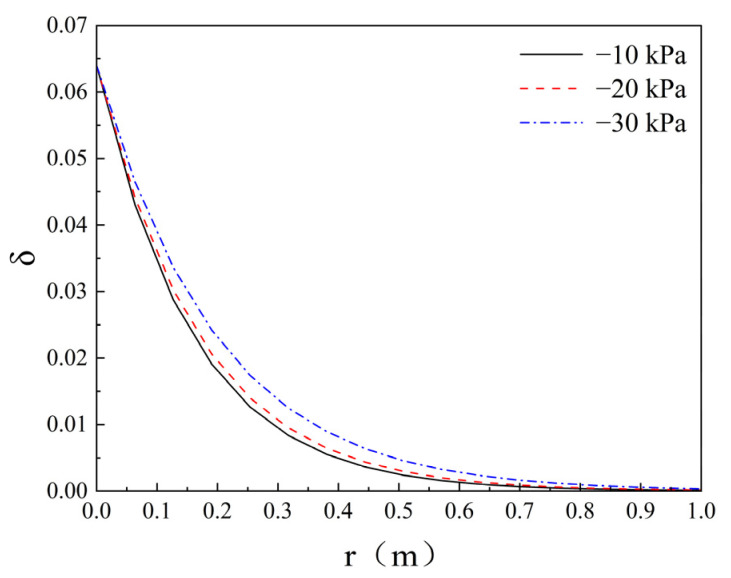
Effect of different vacuum levels on the volume fraction of cement.

Regarding the volume of cement particles leached, the grouting rate may be increased to optimise the permeation grouting effect. Figure 21 illustrates the curve depicting the influence of varying vacuum levels on the cement volume fraction. In terms of the size of cement particle infiltration, the grouting rate can be increased to optimise the effect of infiltration grouting, as shown in Figure 21; according to the calculation of the grouting time and the grouting volume to obtain the grouting rate of ② to ④ groups under different vacuum degrees (−10 kPa, −20 kPa, −30 kPa) were 0.369 × 10^−3^ m/s, 0.395 × 10^−3^ m/s, 0.459 × 10^−3^ m/s, and the rate of grouting was increased by 7.3% and 24.4%, respectively. From Figure 21, it can be seen that the negative pressure of vacuum increases the flow rate of slurry, shortens the retention time of cement particles in the grouting end, and at the same time reduces the porosity attenuation and slows down the attenuation of the permeability coefficient. In conclusion, the vacuum effect significantly inhibits the percolation effect and increases the diffusion distance through the flow rate enhancement and porosity optimisation.

### 5.5. Analysis of the Effect of Injection Pressure

**Figure 22:**
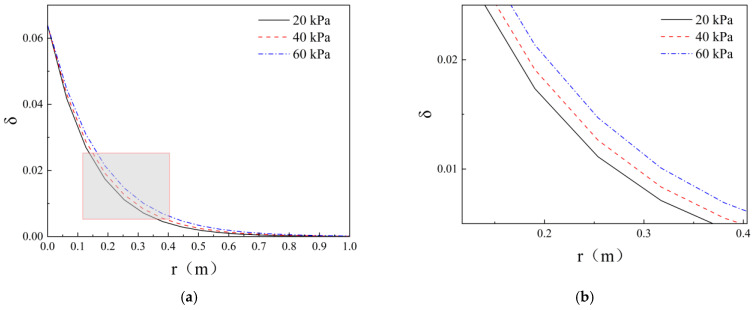
Volume fraction distribution of cement particles under different grouting pressures. (**a**) Overall Curve Chart; (**b**) Localised enlargement.

The increase in grouting pressure will directly push the slurry to penetrate further into the pore space of the formation, especially in the coarse sand layer, sand pebble layer, and other formations with large pore spaces. The pressure increase can significantly expand the diffusion distance. When the pressure exceeds the splitting pressure of the formation, the infiltration diffusion may turn into splitting diffusion, forming cracks and leading to a sharp increase in the amount of slurry absorption. At this stage, the permeation regime transitions from filtration-dominated flow to hydraulic fracturing, resulting in accelerated propagation distance growth due to fracture propagation. However, too high a pressure may lead to premature gelation of the slurry or stratum splitting, instead of reducing the effective infiltration range, but also unfavourable to the more sensitive environment to stress and deformation. From the above analysis, it is evident that grouting pressure is closely related to the effectiveness of permeation grouting. According to the infiltration grouting test results, the grouting rate of ②~④ groups under different grouting pressures (20 kPa, 40 kPa, 60 kPa) was calculated to be 0.344 × 10^−3^ m/s, 0.369 × 10^−3^ m/s, 0.408 × 10^−3^ m/s, and the rate of grouting was increased by 7.3% and 18.7%, respectively. The volume fraction distribution of cement particles at different grouting pressures was plotted, as shown in Figure 22. From Figure 22, it can be seen that the higher the injection pressure and the faster the rate, the volume fraction of cement particles injected into the cement slurry in the porous medium decreases gradually, and the number of cement particles retained decreases, which makes the slurry diffusion distance increase and enhances the effect of infiltration grouting. Unlike increasing the vacuum degree, when the grouting pressure is increased by the same multiple, the increase in the grouting rate is relatively small, which in turn has a relatively small influence on the infiltration effect. When it comes to infiltration grouting, only by increasing the grouting pressure, not only does it increase the risk of causing premature gelation of the slurry or stratum splitting, which in turn affects the stability of the weak soil layer or produces large deformation, but also has a certain degree of limitation on the enhancement of the grouting effect. According to the literature [18], when the vacuum exceeds 60 kPa, slurry pulses may appear in vacuum measurements; therefore, the vacuum should be maintained below −60 kPa. During grouting operations, the injection flow rate should be controlled between 7 and 10 L/min. Grouting pressure within sand layers is typically maintained below 0.3 MPa. Grouting may cease once the injection pressure reaches the design value and remains stable for 15 to 20 min [38].

Finally, we implemented extremely rigorous homogenisation procedures during the experiments. Sand samples underwent strict screening and were prepared at a uniform compaction density (each layer weighing 1.4 kg). Precise measurements were taken during grout preparation, while injection pressure and vacuum levels were meticulously controlled via precision pressure-regulating valves, thereby minimising inherent sample variability to the greatest extent possible. We acknowledge that the experimental uncertainties (such as *p*-values and standard deviations) were not considered, which indeed constitutes a limitation of this study. Consequently, we plan to refine and improve this aspect in subsequent research.

## 6. Conclusions

Based on a combined approach of multi-scale theoretical modelling and experimental validation, this study systematically investigated the coupling mechanisms of seepage and consolidation for cement-based grout within porous media. A macroscopic flow model incorporating particle filtration effects was established based on non-Darcian flow theory. An orthogonal experimental design was employed to optimize the grout mix proportions for superior workability, and the mechanical properties of the optimised mix were characterised. Through vacuum-pressure coupled grouting tests, the study quantitatively elucidated the regulatory mechanisms of vacuum degree and injection pressure on both the filtration effects and the diffusion distance of the WPU-CS. These findings provide a robust theoretical foundation and technical basis for the efficient reinforcement of sandy soil strata. The specific conclusions are as follows:

(1) This paper proposes a suspension flow model in porous media considering the particle percolation effect of suspension in the flow process and applies it to the infiltration grouting of cement slurry in sandy soil. The model depends on two parameters: the volume fraction of cement particles δ of the slurry and the porosity ϕ of the porous medium. The cement particle volume fraction δ of the porous medium. The cement particle volume fraction decreases with increasing distance r from the grouting end, and the front-end velocity of the slurry gradually slows down as the distance from the grouting end increases. The porosity ϕ decreases in the range of penetration grouting, and the closer the distance from the grouting end, the smaller the porosity.
(2) Based on orthogonal experimental analysis of water–cement ratio, polyurethane–to-cement ratio, suspension stabiliser, and water-retaining agent content, the optimal formulation for WPU-CS was determined. The optimal slurry ratio was a water–cement ratio of 1.5:1, poly–ash ratio of 5%~10%, suspending agent content of 1%, and water-retaining agent content of 0.15%.
(3) Compared with pure cement slurry, WPU-CS can significantly inhibit the percolation effect during infiltration grouting and thus enhance the effect of grouting waterstopping reinforcement in the sandy soil layer. Based on the experimental results, increasing both vacuum and grouting pressure accelerates slurry flow, reduces the decay rate and retention of cement particle volume fraction, expands slurry diffusion radius, effectively suppresses filtration effects, and enhances grouting efficiency. However, increasing the grouting pressure only during infiltration grouting may lead to premature gelation of the slurry or cause stratum splitting, which may affect the stability of the weak soil layer and produce large deformation. This study demonstrates that using WPU-CS with optimised grouting parameters—moderate pressure coupled with high vacuum—enables effective control over filtration behaviour while ensuring operational safety. This approach achieves a sustainable equilibrium between grouting efficiency and construction reliability, offering an innovative and practical solution for reinforcing sandy soil strata with significant engineering applicability.

## Figures and Tables

**Table 1:** Superfine cement performance parameter table.

Stability	Heat Loss (%)	Chloride Content (%)	Initial Setting Time (min)	Final Setting Time (min)	Compressive Strength (MPa)		Flexural Strength (MPa)	
					3d	28d	3d	28d
Eligible	5.0	0.06	30	600	23.0	52.5	4.0	7.0

**Table 2:** Parameters of hydroxypropyl methyl cellulose.

Moisture (%)	Viscosity (mPa·s)	Ash (%)	Transmittance (%)	Water Insoluble Matter (%)	Methoxy Content (%)	Gel Temperature (°C)	Hydroxypropyl Content (%)	pH
3%	200,000	3	96	0.3	29	65	13	6.5

**Table 3:** Orthogonal test factor level table.

	Considerations	Water–Cement Ratio (Weight Ratio) A	Water-Based Polyurethane B (%)	Suspensions C (%)	Water-Retention Agent D (%)
Level					
1		1:1	5	0.5	0.1
2		1.5:1	10	1	0.15
3		2:1	15	2	0.2

**Table 4:** Slurry proportioning table.

Test No.	Water–Cement Ratio (Weight Ratio) A	Water-Based Polyurethane B (%)	Suspensions C (%)	Water-Retention Agent D (%)
1	1:1	5	0.5	0.1
2	1:1	10	1	0.15
3	1:1	15	2	0.2
4	1.5:1	5	1	0.2
5	1.5:1	10	2	0.1
6	1.5:1	15	0.5	0.15
7	2:1	5	2	0.15
8	2:1	10	0.5	0.2
9	2:1	15	1	0.1

**Table 5:** Experimental protocol.

Test No.	Degree of Vacuum (kPa)	Grouting Pressure (kPa)	Water-Based Polyurethane (%)	Water–Cement Ratio (Weight Ratio)	Water-Retention Agent (%)	Suspensions (%)	Grouting Time (s)
1	−10	40	0	1.5:1	0	1	100
2	−10	40	5	1.5:1	10	1	100
3	−20	40	5	1.5:1	10	1	100
4	−30	40	5	1.5:1	10	1	100
5	−10	20	5	1.5:1	10	1	100
6	−10	60	5	1.5:1	10	1	100

**Table 6:** Extreme variance analysis of slurry performance test results.

**Water precipitation rate** **(%)**	K1	13.5	14.7	28.5	27.2
	K2	10.7	15.7	15.5	6.7
	K3	23.7	17.5	3.9	14.5
	κ1	4.5	4.9	9.5	9.1
	κ2	3.6	5.2	5.2	2.2
	κ3	7.9	5.8	1.3	4.8
	Range	3.4	0.9	8.2	4.3
	Optimal case	A_3_	B_1_	C_3_	D_2_
**Apparent viscosity** **(mPa·s)**	K1	947	212	123	129
	K2	130	437	414	522
	K3	193	621	733	619
	κ1	315.7	70.7	41.0	43.0
	κ2	43.3	145.7	138.0	174.0
	κ3	64.3	207.0	244.3	206.3
	Range	272.4	136.3	203.3	163.3
	Optimal case	A_2_	B_1_	C_1_	D_1_
**Gel time** **(min)**	K1	18	46	92	73
	K2	84	46	78	66
	K3	91	101	23	54
	κ1	6.0	15.3	30.7	24.3
	κ2	28.0	15.3	26.0	22.0
	κ3	30.3	33.7	7.7	18.0
	Range	24.3	18.4	23.0	6.3
	Optimal case	A_1_	B_1_	C_3_	D_3_

**Table 7:** Extreme variance analysis of the test results of the mechanical properties of the consolidated grout specimen.

**Compressive strength** **(MPa)**	K1	7.9	2.2	2.5	1.2
	K2	2.9	4.8	5.0	5.0
	K3	1.1	4.7	4.3	4.6
	κ1	2.6	0.7	0.8	0.4
	κ2	1.0	1.6	1.7	1.7
	κ3	0.4	1.6	1.4	1.5
	Range	2.2	0.9	0.9	1.3
	Optimal case	A_1_	B_2_	C_2_	D_2_
**Flexural strength** **(MPa)**	K1	5.7	3.1	3.1	1.6
	K2	2.9	3.1	3.8	3.0
	K3	1.2	3.5	2.8	5.2
	κ1	1.9	1.0	1.0	0.5
	κ2	1.0	1.0	1.3	1.0
	κ3	0.4	1.2	0.9	1.7
	Range	1.5	0.2	0.4	1.2
	Optimal case	A_1_	B_3_	C_2_	D_3_

## Data Availability

The original contributions presented in this study are included in the article. Further inquiries can be directed to the corresponding authors.

## Abbreviations

The following abbreviations are used in this manuscript:

WPU-CS	water-based polyurethane-cement composite grout
SEM	scanning electron microscope
WPU	waterborne polyurethane
OPU	oilborne polyurethane
PUMC	polyurethane-modified cementitious mortar
